# Investigating the impact of COVID-19 on performance and image enhancing drug use

**DOI:** 10.1186/s12954-021-00571-8

**Published:** 2021-12-04

**Authors:** Matthew Dunn, Timothy Piatkowski

**Affiliations:** 1 School of Health and Social Development, Deakin University, Geelong Waterfront Campus, Locked Bag 20000, Geelong, VIC 3220 Australia; 2 Queensland University of Technology, Brisbane, Australia

**Keywords:** Steroids, Anabolic–androgenic steroids, COVID-19, Performance and image enhancing drug use, Exercise

## Abstract

**Background:**

Emerging research has suggested that the COVID-19 pandemic has had some impact on substance use patterns. The aim of the study was to conduct a rapid survey to assess the impact of COVID-19 on performance and image enhancing drug (PIED) use and training, and any subsequent negative physical or mental health outcomes.

**Methods:**

During 2020, a convenience sample of 60 PIED consumers (mean age = 26.69; 68.3% located outside Australia) completed a quantitative anonymous online survey exploring how the coronavirus pandemic impacted patterns of PIED use and associated exercise habits. The survey was administered via the Qualtrics platform and distributed online through PIED forums as well as through the investigators’ networks. Participants were asked about their PIED use and exercise habits prior to and during restricted movement ‘lockdowns’.

**Results:**

During pre-COVID, the majority of the sample opted to ‘blast-cruise’ (an initial high dose, followed by a lower maintenance dose; 71.7%, *n* = 43). During lockdown, 45% *(n* = 27) reported a change in PIED use as a result of the restrictions. In light of health concerns during COVID-19, a majority of men (60%, *n* = 36) did not take any extra precautions relating to their PIED use. A subgroup of men ceased using PIEDs completely (16.7%, *n* = 10) with the majority (80%, *n* = 8) of that subgroup following post-cycle therapy (PCT) of some kind. Only a small proportion of the sample reported negative mental health issues as a result of PCT access issues.

**Conclusions:**

This study contributes to the emerging literature of the impact of the COVID-19 pandemic on substance use, specifically PIED use among men. The results suggest that the pandemic did influence the choice of PIEDs that participants consumed, although there was little disruption to patterns of exercise, an important aspect of PIED use. Of the men who did cease use completely, the majority reported little issue with PCT access; those who reported difficulty accessing PCT compounds indicated experience some mental health concerns related to ceasing their PIED use. Clinicians and those who come into contact with this group should be alert for any negative physical or mental health concerns resulting from disrupted or ceased PIED use.

## Background

The COVID-19 pandemic has caused significant worldwide disruption to almost all aspects of daily life. As the SARS-CoV-2 virus began to rapidly circulate around the world, governments of all levels were forced to enact strict interventions aimed at disrupting the virus’s spread, including closing borders, shutting businesses, and restricting social interactions. Many of these restrictions occurred with little notice to the population, with travellers caught stranded in foreign countries as borders closed; businesses orienting to online sales; schools and universities pivoting to online learning; and some industries shutting completing. Faced with disruptions of indeterminant length, populations were forced to quickly adapt. The shift to working at home, as well as increased stress, led to concerns about an increase in the purchase and consumption of alcohol [1]. However, since the commencement of the pandemic, there has been a concentrated focus on anticipated changes in illicit substance use. There is good reason for this; drug markets can be disrupted by significant events [2], with changes in price, purity, and availability influencing the types of drugs used, and the patterns in which they are used. This in turn can have an impact on the harms experienced, as well as help seeking behaviour.

Emerging research has suggested that the pandemic has had some impact on substance use. Research with frequent psychostimulant uses in Australia found that consumers reported either no change or a reduction in their substance use, with reductions in MDMA use mostly related to reduced opportunities to socialise [3]. An audit of illicit drug-related presentations to an emergency department found a decrease in presentations during COVID compared with pre-COVID [4]. Greater concern has been given to those already living with a substance use disorder and in some form of treatment, given the disruption caused to accessing treatment and harm reduction services; however, some research suggests that this group may have experienced fewer consequences than anticipated; one Austrian study with patients in opioid substitution treatment found that while this group did experience difficulties, these were less severe than expected [5]. It is important to recognise that in all instances, these findings reflect the differing situations in the countries where the research was conducted, as well as the specific populations under investigation. For instance, a study exploring the impact of lockdowns and social distancing on people who use drugs by analysing social media posts found that these restrictions led to some people experiencing forced or intentional withdrawal, as well as impacting their ability to access take-home medication [6].

Of interest to the current study was the impact of mass restrictions on the use of performance and image enhancing drugs (PIEDs), the collective term used to refer to substances which are used to enhance sporting or athletic performance and/or for enhancing body image. The most well-researched PIED is anabolic–androgenic steroids (AAS). These are a group of hormones that possess both anabolic properties, which causes muscle growth and fat loss, and androgenic properties, which causes such masculinising effects as voice deepening and facial hair growth. They have also been used non-medically for several decades in bodybuilding, fitness, and sporting populations for their growth promoting-properties. Unlike other recreationally used substances (e.g. MDMA, ‘ecstasy’), the effects of most PIEDs are not felt instantly; they often take several weeks to be metabolised by the body and for the desired effects to be noticed. In the case of AAS, the body shuts down its own testosterone production in the presence of exogenous testosterone, which may take several weeks. Consequently, it may take some time, up to several years, for the body to start producing testosterone again once a person ceases exogenous AAS use. Due to this lag time, people who use these substances will plan their use to coincide with a time when the desired effects will be optimal, such as a bodybuilding competition. A specific regime of substances may be used to help lose body fat and to promote muscle growth, and to assist the body to ‘kick start’ its own production of these hormones once this regime is finished. This process of restarting the body natural hormone production is generally referred to as post-cycle therapy (PCT) [16]. PCT is an important feature of PIED use and refers to the period when the consumer ceases PIED use (also known as an ‘off cycle’). Consumers may access PCT for a variety of reasons, such as to minimise any loss of muscle or strength gained through their PIED cycle; because they are concerned that they were no longer naturally producing hormones; or because they are concerned about their mental health, particularly when coming ‘off cycle’ [7].

Given the nature of how PIEDs are used, the closure of borders, restrictions of movement and social interactions, as well as the closure of many gyms and other fitness institutions may have impacted PIED supply and use. Most PIEDs are purchased from those known to the consumer [8], such as friends and others in the fitness industry, and most PIEDs are manufactured from raw materials which are purchased from countries such as China. Border closures may have disrupted drug importations; research investigating border seizures in the USA found an immediate decrease in cannabis and methamphetamine seizures in April 2020 which then increased in August 2020 [9], and research suggests that there were also disrupted illicit drug transactions on the dark web [10], which are still impacted by normal market forces [11]. As such, PIED consumers may have had their supply disrupted with little or no notice, which could lead to negative physical and mental effects.

Since the commencement of the pandemic, there has been an emerging body of literature exploring the impact of the pandemic on PIED use. Zoob Carter, Boardley & van de Ven [12] found that strength athletes using non-prescribed AAS perceived an impact of the pandemic on their AAS use and training, with significant reductions in training frequency and AAS dose compared to pre-pandemic, and while there were some short-term effects on mental health, these did not appear to be long lasting. In their study of exercise and enhancement drug use in seven countries, Dores et al. [13] found that 4% of the sample engaged excessive or problematic exercise during lockdown. While 28% reported lifetime PIED use, 19.7% of those reported maintained their use during lockdown, with only 1.5% ceasing use. It should be noted, however, that much of this use was of vitamins, proteins, and other legally available PIEDs, with only a small proportion (1.4%) reporting use of androgenic substances. That study also found a strong association between physical activity and PIED use, and anxiety about appearance significantly increased the probability of using PIEDs. The aim of the present study was to explore the impact of lockdowns on PIED use and physical activity, and any subsequent negative physical or mental health outcomes. Specifically, the aims were to characterise PIED use pre- and during lockdowns; to characterise physical activity practices pre- and during lockdowns; to ascertain whether lockdowns had disrupted the supply of and access to PIEDs; whether any difficulty accessing PIEDs had to lead to cessation of use and, if so, whether this resulted in any negative physical or mental health outcomes.

## Methods

### Procedure

An online survey was used explore how the coronavirus pandemic impacted peoples’ exercise habits and their use of PIEDs. A convenience sample was recruited via advertisements on various social media platforms, such as bodybuilding and PIED-related subreddits on reddit.com and other online forums that were dedicated to those topics. The study was also advertised in the ‘Drug Studies’ thread on the Bluelight.org website. A link to the survey was posted on the authors’ social media accounts, as well as forwarded to author contacts within the fitness industry. The second author also discussed the survey on several podcast interviews, and a link to the survey was available in those episodes’ show notes. The advertisement outlined the purpose of the study and invited those who use PIEDs such as AAS (e.g. Testosterone, Nandrolone, Trenbolone), peptides (GHRP-6, Hexarelin, IGF-1), and hormones (Human growth hormone, insulin) primarily to enhance performance and/or appearance through promoting muscle gain and/or fat loss to complete a short survey, with the link provided to the main survey page. This page contained more detailed information about the study, including all relevant ethical information and the consent process. Apart from the eligibility criteria stated above, an additional criterion was that people under the age of 18 years of age should not participate. The survey was available from July 2020 to December 2020; 94% of responses were collected between September and November 2020.

After confirming their consent, participants completed the survey which contained four sections:

‘Background’ section contained brief demographic questions, including gender, age, sexual identity, weight, height, and whether the participant lived within or outside Australia.

‘Methods’ section contained questions about exercise habits in the four weeks prior to the COVID-19 lockdown (with participants being told that ‘lockdown’ referred to the ‘instance where most business, including gyms, were required to shut by their Government in response to the COVID-19 pandemic’), including the type of exercise training the participant was primarily engaged in (both weight training and cardio, but primarily weight training; both weight training and cardio, but primarily cardio; weight training with minimal/no cardio; cardio with minimal/no weight training), frequency of training per week, location of training (home gym, friend’s home gym, commercial gym, private studio, outdoor training), whether the participant trained by themselves or in a group, and whether the participant was training to add muscle mass or to cut body fat.

‘Results’ section contained questions about the participant’s normal PIED use, including whether the participant was engaged in ‘cycling’ steroids or ‘blast and cruise’, how long their on and off cycles were if they were cycling, what injectable and/or oral steroids they were consuming (with a list provided and space for the participant to note any that were not listed), and how they usually acquired their PIEDs (from a friend, a dealer, websites on the Internet, websites on the dark web, from a veterinarian, or other); and ‘Discussion’ section contained questions about exercise habits and PIED use during lockdown, including the type of exercise training the participant was primarily engaged in, whether intensity of training had changed, frequency of training per week, location of training (home gym, friend’s home gym, commercial gym, private studio, outdoor training), whether the participant trained by themselves or in a group, whether PIED use had changed as a result of restrictions, whether there had been changes in the cost or quality of the PIEDs the participant used, any PCT regimes the participant engaged in, and whether the participant had experienced any negative physical or mental health effects as a result of changes in PIED use and if so what these were.

The survey was pilot tested prior to launch by two consumers known to the second author. Minor alterations were made, such as the inclusion of some demographic questions. The survey was designed to collect pertinent information about the topic in a short amount of time (hence our referral to it as a ‘rapid survey’). The survey took approximately 5–10 min to complete. All responses were self-reported.

Participants in Australia who completed the survey were offered the opportunity to go into the draw for one of five AUD$20 gift vouchers for an online supplement supplier.

### Data analysis

Data were exported from the Qualtrics platform and analysed using IBM SPSS Statistics 27. Initially, the data consisted of 87 participants; however, 27 of these were incomplete or had upwards of 60% data missing at random on key variables. There was no data imputation used. Percentages are presented for categorical variables and means or medians presented for continuous variables. Categorical variables were analysed using Chi-square tests, with odds rations (ORs), 95% confidence intervals (95% CIs) and *p* values reported where tests were significant. All analyses were conducted using SPSS for Windows, Version 26.0.

Ethical approval was granted by the Deakin University Human Research Ethics Committee.

## Results

Participants were 60 men (Mean age = 26.69, SD = 6.57) who identified as primarily heterosexual (95%, *n* = 57) with the remainder identifying as bisexual (3.3%, *n* = 2) or queer (1.7%, *n* = 1). A large portion of the sample resided outside Australia (68.3%, *n* = 41), with the remainder living within Australia (31.7%; *n* = 19). Seven men (11.7%) had competed in a bodybuilding competition. Sample characteristics are provided in Table 1 for all participants, as well as a comparison between those who were cycling and those who were blast-cruising. There were no significant differences between these groups of demographic variables.

**Table 1:** Participant characteristics and types of PIEDs used prior to lockdown

	Sample	Blast-Cruise		OR*[t]*	95% CI	*p* value
	(*n* = 60)	No (*n* = 17)	Yes (*n* = 43)			
Mean age	26.69	27.56	26.37	[.616]	-	n.s
Mean BMI	26.20	25.27	26.20	[1.779]	-	n.s
Mean percentage body fat	15.00	15.60	15.13	.119	-0.5,0.6	n.s
Competed in a bodybuilding show (%)	11.7	94.5	86.7	0.4	0.1,3.5	n.s
Heterosexual (%)	95.0	94.5	95.4	1.3	0.1,15.1	n.s
Injectable (%; *n*)						
Testosterone	70; 42	47.1	79.1	4.3	1.3,14.2	0.007
Trenbolone	28.3; 17	5.9	37.2	9.5	1.1,78.4	0.007
Boldenone	23.3; 14	11.8	27.9	2.9	0.5,14.7	n.s
Drostanolone	16.7; 10	5.9	20.9	4.2	0.5,36.4	n.s
Stanazolol	13.3; 8	6.3	14.0	2.6	0.3,23.3	n.s
Nandrolone	13.3; 8	11.8	16.3	1.5	0.3,7.8	n.s
Methenolone	5; 3	11.8	2.3	0.2	0.1,2.1	n.s
Oral (%; *n*)						
Oxandrolone	28.3; 17	17.6	32.6	2.3	0.6,9.1	n.s
Metandienone	25; 15	17.6	27.9	1.8	0.4,7.4	n.s
Oxymetholone	13.3; 8	5.9	16.3	3.1	0.4,27.4	n.s
Stanazolol	11.7; 7	5.9	16.3	3.1	.4,27.4	n.s
Mesterolone	8.3; 5	5.9	9.3	1.6	0.2,15.8	n.s
Chlorodehydromethyltestosterone	1.7; 1	5.9	0.0	0.3	0.2,0.4	n.s
Fluoxymesterone	1.7; 1	0.0	2.3	0.7	0.6,0.8	n.s
Other PIEDs (%; *n*)						
Clenbuterol	16.7; 10	5.9	20.9	4.2	0.5,36.4	n.s
Human-growth-hormone	10; 6	5.9	11.6	2.1	0.2,19.5	n.s
Peptides	10; 6	11.8	9.3	0.8	0.1,4.6	n.s
Insulin	8.3; 5	5.9	9.3	1.6	0.2,15.8	n.s
Prohormones	8.3; 5	0.0	11.6	0.7	0.6,0.8	n.s
Selective-androgen-receptor-modulators	6.7; 4	5.9	7.0	1.2	0.1,12.4	n.s
T3	5; 3	5.9	4.7	0.8	0.1,9.2	n.s
Insulin-like-growth-factor-1	5; 3	5.9	4.7	0.8	0.1,9.2	n.s

Specific *p* values available from authors on request

### Activities pre-COVID

The men in this sample were generally involved in weight training (*n* = 59) with one participant being primarily involved in cardiovascular training (*n* = 1). The most common training volume for these men was four or more days per week (95%, *n* = 57) which occurred at either a commercial gym (51.7%, *n* = 31), home gym (23.3%, *n* = 14), a local non-commercial gym (15%, *n* = 9), outdoor training (5%, *n* = 3) private studio (3.3%, *n* = 2), or a friend’s home gym (1.7%, *n* = 1). Most men in this group opted for training by themselves (91.7%, *n* = 55) rather than a group (8.3%, *n* = 5), with some men reporting having a coach/personal trainer (13.3%, *n* = 8) and training/preparing for a competition (20%, *n* = 12).

With respect to PIED use, a majority of the sample opted to ‘blast-cruise’ (an initial high dose, followed by a lower maintenance dose; 71.7%, *n* = 43) as opposed to ‘cycling’ (using a dose for a defined period, then ceasing to use for a defined period; 28.3%, *n* = 17). Regarding the actual PIEDs used, these are presented in Table 1. Those who reported using PIEDs in a ‘blast-cruise’ regime were significantly more likely to report the use of testosterone (79.1% vs. 47.1%; OR = 4.3; 95%CI 1.3, 14.2; *p* = 0.007) and trenbolone (37.2% vs. 5.9%; OR = 9.5; 95%CI 1.1, 78.4; *p* = 0.007) prior to lockdown than those who did not; no other significant differences were found (Table 1).

In acquiring these compounds, a large proportion opted to do so online (51.7%, *n* = 31) and the darkweb (10%, *n* = 6), with 18.3% (*n* = 11) sourcing from a friend, 13.3% (*n* = 8) sourcing from a dealer, and 15% (*n* = 9) sourcing through other means. A small subgroup of men were tapering these compounds in preparation for an upcoming bodybuilding show (8.3%, *n* = 5).

### Activities during COVID

During the COVID-19 restrictions, there were some shifts in the sample’s general activity. One-third (31.7%; *n* = 19) reported that their training decreased during lockdown, with 15% (*n* = 9) reporting that it increased and 13.3% (*n* = 8) reporting that it stayed the same; 24 respondents did not answer this question. Almost one-fifth (16.7%; *n* = 10) stopped training altogether, with an increasing representation of cardiovascular activity among the sample (18.4%, *n* = 11). There was a minor shift in the number of days those men were training with a larger proportion reporting under 4 days (13.4%, *n* = 8), while the remainder reported between 4 (6.7%, *n* = 4) and 7 (8.3%, *n* = 5) days of training; the largest proportion was still training between 5–6 days (25%, *n* = 15). The men that continued to train reported predominantly doing so in a home gym (35%, *n* = 21). This was not significantly different between those cycling or those engaging in a ‘blast-cruise’ regime.

More than two-fifths (45%; *n* = 27) indicated that they had changed their PIED use, with three indicating that this was due to difficulty accessing compounds. A subgroup of men ceased using PIEDs completely (16.7%, *n* = 10) with the majority (80%, *n* = 8) of that subgroup following PCT of some kind. Of this subgroup a large proportion reported their use of alcohol and other drugs to change (80%%, *n* = 8). Half of the subgroup (50%, *n* = 5) reported issues accessing PCT compounds which were predominantly associated with peer networks (*n* = 1) or peer networks and GP refusal (*n* = 4). A small number of this subgroup (30%, *n* = 3) reported experiencing negative health effects as a resulting of PIED cessation. Two participants reported the common issues which arise as a result of sudden AAS cessation—e.g. depressed mood, little energy, and low libido. One participant expressed that their mental health declined significantly and experienced a number of symptoms such as depression, lethargy, anxiety, panic attacks, and suicidal ideation.

A majority of men (60%, *n* = 36) did not take any extra precautions relating to their PIED use (e.g. sterilising vials, not reusing needles) although some reportedly did (16.7%, *n* = 10). In terms of changes around compound access and sourcing, a majority reported no change in price (60%, *n* = 36) or quality (56.7%, *n* = 34). Lastly, 53.3% (*n* = 32) reported no issues with accessing needle and syringe programs.

There were no significant differences between those who engaged in a ‘blast-cruise’ regime and those who were not with regard to physical activity pre-lockdown and during lockdown. However, those who engaged in a ‘blast-cruise’ regime were significantly less likely to report ceasing their PIED use during lockdown (30.2% vs. 70.6%; OR = 0.2; 95%CI 0.1, 0.6; *p* = 0.002); of those who did cease their PIED use during lockdown, those who engaged in a ‘blast-cruise’ regime were significantly less likely to self-report experiencing negative health effects (4.7% vs. 29.4%; OR = 8.5; 95%CI 1.5, 6.2; *p* = 0.003) (Table 2).

**Table 2:** Exercise and PIED use patterns pre-lockdown and during lockdown

Variable	Sample	Blast Cruise		OR [t]	95% CI	*p* value
	(*N* = 60)	No (*n* = 17)	Yes (*n* = 43)			
Pre-lockdown (%; *n*)						
Weight-training	98.3; 59	100	95.4	1.4	1.2,1.7	n.s
Train at a gym	95.0; 57	88.2	95.4	5.6	0.5,6.3	n.s
Home gym	25; 15	5.9	32.6	7.7	0.9,64.3	0.001
Train by self	91.7; 55	100	88.4	1.4	1.2,1.7	n.s
During lockdown (%; *n*)						
Weight-training	83.3; 50	88.2	88.4	1.4	1.3,2.4	n.s
Train at a gym	88.9; 32	100	85.7	1.3	1.1,1.6	n.s
Home gym	35; 21	29.4	37.3	1.4	0.4,4.8	n.s
Train by self	80.6; 29	100	75.0	1.4	1.1,1.7	n.s
PIED use during lockdown (%; *n*)						
PIEDs use changed	45.0; 27	23.5	34.9	1.7	0.5,6.3	n.s
Change due to access issues	60.0; 36	58.8	60.5	1.1	0.3,3.6	n.s
PIEDs precautions (e.g. more swabbing)	40.0; 24	52.9	34.9	0.5	0.2,1.5	n.s
PIEDs Cessation	16.7; 10	70.6	30.2	0.2	0.1,0.6	0.002
If ceased, used post-cycle therapy	13.3; 8	11.8	4.7	2.6	0.2,7.7	n.s
If ceased, had negative health effects	5.0; 3	11.8	2.3	8.5	1.5,6.2	0.003
Issues in accessing harm reduction	46.7; 28	52.9	44.2	0.7	0.2,2.2	n.s
Acquisition of PIEDs online	51.7; 31	41.2	55.8	1.8	0.6,5.6	n.s

Specific *p* values available from authors on request

## Discussion

The COVID-19 pandemic has disrupted almost all facets of daily life. Of concern has been how this pandemic has shifted substance use habits. Physical activity, and specifically weight training, is psychologically and physically beneficial [14, 15], and a key concern during lockdown periods has been the negative health effects that may be experienced by those who regularly engage in fitness and may not be able to access the facilities to do so. The focus of this study was to explore whether the initial restrictions imposed on movement, such as the closure of gymnasiums, impacted PIED use. Given that PIED use often occurs within a strict diet and fitness regime, it could be hypothesised that the shutting of gyms and other fitness facilities may have had a flow-on effect by impacting PIED use. Further, given that social supply is common with PIED-consuming communities [8, 16], the lack of social interaction during lockdowns may have disrupted this social supply, potentially causing some consumers to cease use, which in turn may have had negative physical and mental health effects. The findings of this study suggested that among this sample, lockdowns did not have an impact on training or PIED use—only one-third decreased their training volume and two-fifths changing their PIED use, with only a small proportion of those indicating that this was due to difficulties in accessing PIEDs. While a small proportion ceased PIED use altogether during their lockdown period, most of those start on a regime of PCT. Only three participants from the whole sample reported experiencing negative effects from ceasing their PIED use. Given the possibility that AAS use is linked to COVID-19 disease severity [17], understanding more about this group is of importance.

The findings from this study differ slightly from those reported by Zoob Carter, Boardley & van de Ven [12], whose study found that that strength athletes using non-prescribed AAS perceived an impact of the pandemic on their AAS use and training, with significant reductions in training frequency and AAS dose compared to pre-pandemic. In the current study, less than half of the participants indicated that restrictions impacted their PIED use, and only three participants indicated that this was because they could not access compounds due to supply issues; furthermore, there did not appear to be any shifts in training due to restrictions. It is unclear why the group that changed their AAS did so if it was not due to accessing compounds, although there may be other market factors influencing use. For instance, while sizable proportions of the sample indicated that there had been no change in AAS price, purity, or access to injecting equipment, it may be that others did experience changes in one of more of these and other factors that could have influenced some change in their use. It could also be that training factors dictated a change in AAS use, while participants in this sample largely kept training, there was a shift from anaerobic to aerobic exercise. Again, it is unclear why this occurred, as participants did not indicate that they had difficulty accessing training facilities, and were able to shift to home gyms or outdoor exercise. It could be that as social interactions became limited, those who were reliant on social supply networks for access to substances had some difficulty procuring those substances. Our finding that those engaging in a blast-cruise regime were significantly less likely to report ceasing their PIED use during lockdown does perhaps suggest that those who cycle their use may have only had sufficient compounds to last for their on-cycle period. This is pure speculation on the authors’ behalf, and future research may wish to investigate this in detail.

A subgroup of participants did report cessation of PIED use completely, although a majority of these men followed PCT of some kind and did not report experiencing harms. Some men reported issues with PCT access and, therefore, reported some mental health issues and harm associated with mood changes related to hormone fluctuation. Clinicians and other healthcare providers, including general practitioners, should be aware of the negative effects that may arise from ceasing PIED use, especially if the consumer lacks access to PCT. The current data tentatively suggests that lack of PCT was related to negative health outcomes. As many countries move in and out of lockdowns in the coming years due to outbreaks of coronavirus or responding to new variants of the virus, healthcare practitioners may wish to engage in discussions with their PIED-consuming clients about how they may manage any future disruptions to their PIED use, and whether PCT could be useful in these situations.

The lack of impact of the COVID-19 pandemic on PIED use aligns with other trends in the alcohol and drug space. The data supports the notion that COVID-19 did not impact PIED access as a result of increased utilisation of online and dark web networks by consumers. Furthermore, participants reported negligible changes in price and purity/quality of compounds. The fact that a majority of men continued using PIEDs speaks to the prevalence of online markets in facilitating access to these compounds. In this way, the ease of access highlights a pseudo-protective effect by allowing the men in this study to maintain cruising doses of AAS, perhaps placing less pressure to restructure their compound use entirely which, in turn, may mean addressing underlying issues of their PIED use—reportedly best done in an environment conducive to harm reduction and not subject to external pressures [18].

### Limitations

There are limitations to the current study. Firstly, the online nature of project lends itself to response bias, in that those who completed the survey may be different to those who did not. The characteristics of the sample suggest that this is a group highly engaged in the PIED consuming and fitness/bodybuilding community, who may differ from those who are less engaged in PIED use or those who train less frequently. That is not surprising, given the recruitment method, but it does suggest that perhaps this is a group for whom disruptions to general supply chains or the closure of gymnasiums poses little disruption to their individual circumstances. For those who rely on purchasing PIEDs from a close contact, such as a personal trainer or a friend, restrictions may have posed more of a challenge. Secondly, the sample size is lower than what the research team had anticipated. Recruitment was done through a variety of avenues, such as through personal contacts as well as a variety of online forums. Recruitment did occur commence in July 2020, when many countries were undergoing severe disruption, and it could just be the case that participants were concerned more with the daily disruptions occurring in their lives. Research may wish to explore this period of time and the impact of lockdowns on PIED use now that countries are starting to open up and recruitment may be more fruitful.

## Conclusions

This study contributes to the emerging literature of the impact of the COVID-19 pandemic on substance use, specifically PIED use among men. The results suggest that the pandemic did influence the choice of PIEDs that participants consumed, although there was little disruption to patterns of exercise, an important aspect of PIED use. Of the men who did cease use completely, the majority reported little issue with PCT access; those who reported difficulty accessing PCT compounds indicated experience some mental health concerns related to ceasing their PIED use. Clinicians and those who come into contact with this group should be alert for any negative physical or mental health concerns resulting from disrupted or ceased PIED use.

## Data Availability

The data analysed during the current study are available from the corresponding author on reasonable request.

## Abbreviations

AAS: Anabolic–androgenic steroids
GHRP-6: Growth hormone-releasing peptide 6
IGF-1: Insulin-like growth factor 1
PIEDs: Performance and image enhancing drugs
